# Wnt/*β*-Catenin Signaling Pathway in Skin Carcinogenesis and Therapy

**DOI:** 10.1155/2015/964842

**Published:** 2015-05-19

**Authors:** Jing Li, Ling Ji, Jieping Chen, Wengeng Zhang, Zhijia Ye

**Affiliations:** ^1^Institute of Tropical Medicine, Third Military Medical University, 30 Gaotanyan Street, Chongqing 400038, China; ^2^Department of Hematology, Southwest Hospital, Third Military Medical University, 30 Gaotanyan Street, Chongqing 400038, China; ^3^Center for Engineering in Medicine, Department of Surgery, Massachusetts General Hospital, Harvard Medical School, 51 Blossom Street, Boston, MA 02114, USA

## Abstract

Cooperating with other signaling pathways, Wnt signaling controls cell proliferation, morphology, motility, and embryonic development destination and maintains the homeostasis of tissues including skin, blood, intestine, and brain by regulating somatic stem cells and their niches throughout adult life. Abnormal regulation of Wnt pathways leads to neoplastic proliferation in these tissues. Recent research shows that Wnt signaling is also associated with the regulation of cancer stem cells (CSCs) through a similar mechanism to that observed in normal adult stem cells. Thus, the Wnt/*β*-catenin signaling pathway has been intensively studied and characterized. For this review, we will focus on the regulation of the Wnt/*β*-catenin signaling pathway in skin cancer. With the important role in stemness and skin CSC proliferation, the Wnt/*β*-catenin signaling pathway and its elements have the potential to be targets for skin cancer therapy.

## 1. Introduction

Drs. Nusse and Varmus identified the protooncogene integration-1 in 1982 [1]; then int-1 was found to be the mammalian homolog of the segment polarity gene in wingless* Drosophila* (wg) [2]. The name “Wnt” is thus a combination of the two terms, int and wg [3].

In humans, Wnt proteins are a family of 19 lipidated and glycosylated proteins. This type of glycosylated protein initiates signaling through binding with the N-terminal extracellular cysteine-rich region of the Frizzled family (a seven-span transmembrane receptor) and with either LRP5 or LRP6 (two members of the low-density-lipoprotein receptor-related protein family) proteins [4, 5]. Wnt proteins modulate several major signaling cascades including canonical (*β*-catenin-dependent) and noncanonical (*β*-catenin-independent) Wnt signaling pathways. In the canonical Wnt signaling pathway, *β*-catenin is stabilized and translocated to the nucleus, and then it stimulates the target genes and controls gene expression by cooperating with other signaling receptors [6]. In the noncanonical Wnt pathway, *β*-catenin is not activated; instead, signals are transmitted to impact cell polarity and migration through the planar cell polarity pathway and small GTPase proteins [3, 4].

Several proteins interacting with Wnt/*β*-catenin signaling pathways have been identified, such as plakoglobin, a new protein that functions similarly to the *β*-catenin and thus is an important therapeutic drug target. In addition, more and more evidence supports that Wnt/*β*-catenin signaling functions in skin stem cells through cooperation with Cdc42, Notch, and vitamin D [7]. Thereby, the Wnt/*β*-catenin signaling pathway can be controlled by regulating Cdc42, Notch, and vitamin D.

## 2. Wnt/***β***-Catenin Signaling Pathway in Skin Stem Cells

**Figure 1:**
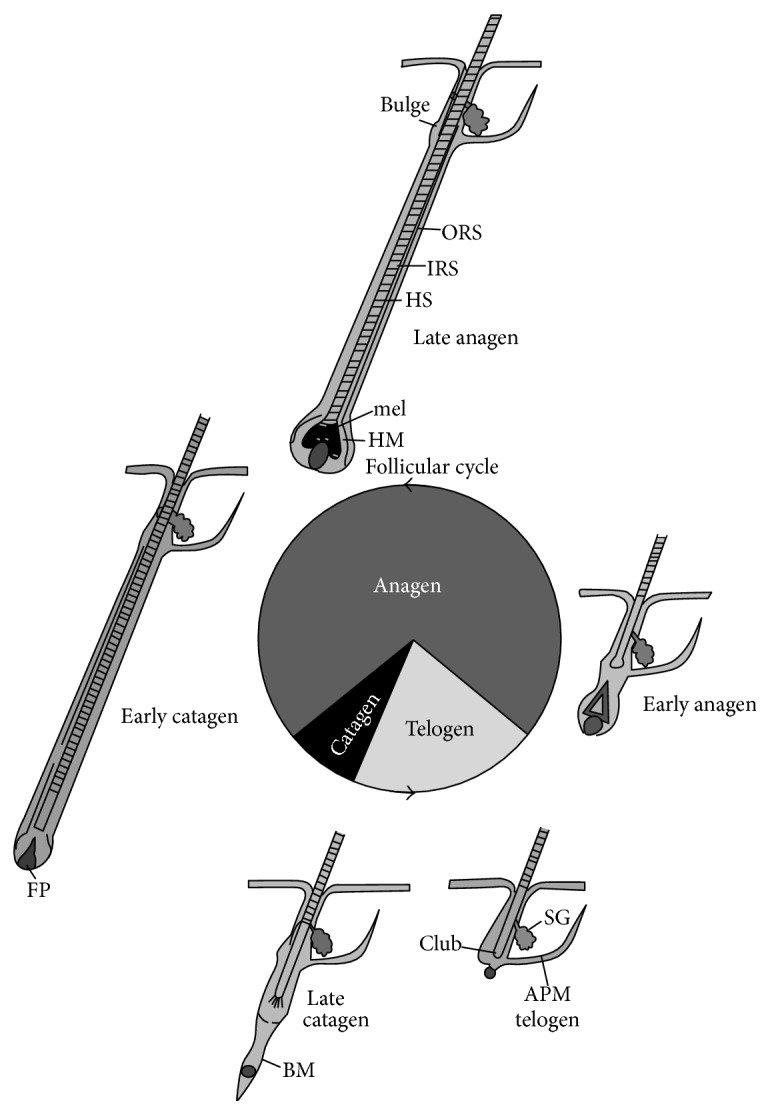
Hair follicle cycling. The initial developmental phase of hair follicle development terminates with catagen and the first telogen, after which repetitive cycles of anagen (the growth phase), catagen (the regression phase), and telogen (the resting phase) occur throughout the life span of the animal. In general, the hair follicle spends most of its time in anagen, but cycle duration varies according to location, gender, age, and species. The bulge is the source of stem cells for the regenerating hair follicle that is responding to signals from the dermal or follicular papilla (FP). ORS: outer root sheath, IRS: inner root sheath, HS: hair shaft, mel: melanin for the hair shaft, HM: hair matrix, BM: basement membrane, SG: sebaceous gland, and APM: arrector pili muscle [8].

There are three populations of skin stem cells in humans. They are located in epidermal proliferative units (EPUs) where basal layer cells are generated, hair follicles (HF) including anagen and telogen follicles, and the bulge where hair follicle stem cells are located (Figure 1) [8].

The epidermis forms the outermost layer of mammalian skin. Cells within the basal layer of the epidermis are the progenitors of the epithelium, which constantly proliferate to ensure renewal of the epithelium. Except for the basal layer, the bulge is an important niche for skin stem cells; the bulge is attached to the hair follicles (HFs). Many Lgr6+ cells reside above the bulge area and have been demonstrated to represent the ability to generate all cell lineages of skin [9]. Almost all types of skin come from the bulge, such as hair follicles and glands. Moreover, bulge stem cells express the stem cell marker CD34 and contain label-retaining cells, such as slow cycling cells. In addition, bulge cells can participate in tissue repair after wounding [3].

Evidence supports that the Wnt/*β*-catenin signal controls the fate of the bulge stem cells and plays an essential role in hair but not epidermal differentiation. Using cre/loxP recombining system driven by the cytokeratin (K)14 promoter to induce the conditional mutation of *β*-catenin in mice, Peter Wend's Laboratory demonstrates that the epidermis of young mutant animals develops normally but without hair [10]. However, there are unusual cyst-like structures appearing in the skin of these mice. Meanwhile, it is found that multilayered epithelium surrounds the cysts [11–13]. At the same time, there is another similar phenotype, with a lack of hair but containing stem cells; however, these cells can only differentiate into epidermal cells but not into hair follicles. Moreover, after ectopic expression of Wnt inhibitor Dkk1 in the skin, it is observed that transgenic expression of dominant-negative Lef1 results in the formation of cysts and other epidermal appendages, such as teeth and mammary glands. A study using cultured epidermal keratinocytes in which a loss-of-function mutation in Wnt/*β*-catenin is introduced by Adenovirus-mediated gene transfer of cre/loxP system demonstrated that Wnt/*β*-catenin plays a minor role in epidermal but not in hair development [14].

In the epidermis, mesenchymal stem cells are self-renewing and differentiate into hair follicles (HFs) when proper stimulations are performed. During this process, signals that are initiated from the dermis can induce epithelium thickening, elongation of epithelial cells, and formation of placodes that contain Wnt-responsive cells [15–17]. In response, placodes signal dermal cells to condense to form the dermal papilla component of the HF, which is also responsive to Wnt signaling [17]. The fully formed HF then retains detectable levels of *β*-catenin in the dermal papilla and matrix cells in which *β*-catenin contributes to the maintenance of HF structures [17, 18].

Neural crest cells represent a transient and highly migratory embryonic cell population, and neural crest stem cells (NCSCs) are characterized by their multipotency and self-renewal abilities [19]. It is demonstrated that NCSCs can renew and differentiate into different types of cells including glia, neurons, smooth muscle cells, adipocytes, osteoblasts, chondrocytes, and melanocytes during embryonic development. It is generally believed that NCSCs exist only in the developing embryo. However, Kruger et al. have found that there are cells with neural crest features similar to embryonic NCSCs in adult gut [20]. This finding initiated numerous studies that have demonstrated the presence of postmigratory NCSCs in a variety of adult organs such as skin [21–26].

Although a substantial number of reports have demonstrated the existence of NCSCs in adult tissues, the physiological role of these cells still remains unknown. Recently, for the first time, a study by Johnston's group [27] demonstrated that neural crest precursor cells could function in wound healing in mature mouse skin. Previously, Johnston et al. identified that multipotent, self-renewing cells reside in adult skin, and then they named these stem cells skin derived precursor (SKP) cells [21]. Subsequent research demonstrated that SKP cells have neural crest-like features, such as expressing neural crest markers and having the same differentiation potential as NCSCs cells [22]. A study using a genetic lineage tracing method in* Wnt1-Cre R26R* mice found that SKP cells were the same as the cells derived from the neural crest lineage [22]. Moreover, the presence of innervation is necessary for organ regeneration in amphibians [28], and neural crest-derived Schwann cells can provide signals for successful tissue repair. After these discoveries, Johnston et al. demonstrated that nerve terminal-associated cells can express the transcription factor Sox2 by cooperating with other markers of neural crest cells, including p75NTR, nestin, and S100 b [27]. After wounding, some Sox2-positive cells started to proliferate and migrate into the regenerating dermis. Moreover, knocking Sox2 out can lead to a significantly decreased rate of wound closure, which proves that Sox2-positive neural crest-derived cells play an essential role in the successful repair of injured skin.

Wnt/*β*-catenin signaling plays a critical role in driving neural crest cells toward a melanocyte cell fate through regulating the homeobox gene microphthalmia transcription factor (MITF) [29–32]. It is observed that nuclear *β*-catenin is in the majority of benign nevi; however, there is no nuclear *β*-catenin in melanoma progression [33–35]. Thus, activation of Wnt/*β*-catenin signaling may be essential for cellular homeostasis. Consequently, a loss of Wnt/*β*-catenin signaling may result in the dysregulation of specific transcriptional programs in melanocytes and nevus cells, which may lead to lower survival rates in melanoma patients [33–35].

## 3. Wnt/***β***-Catenin Signaling Pathway Crosstalk with Other Signaling in Skin Stem Cells

**Figure 2:**
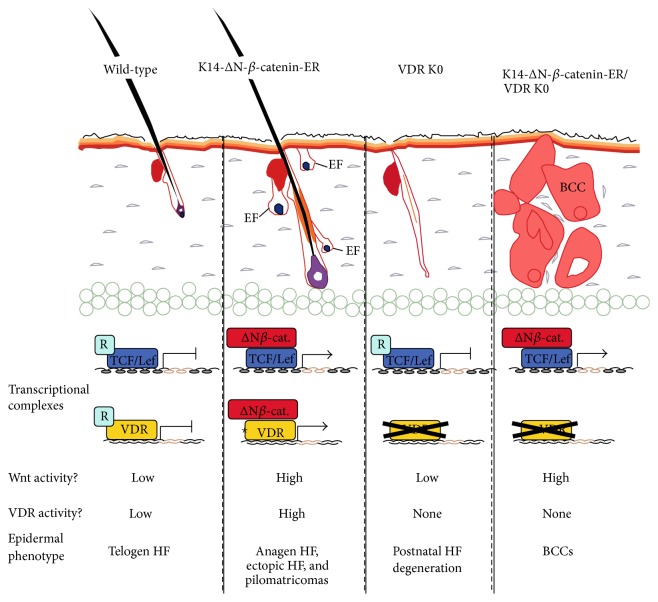
Interaction among *β*-catenin, Lef/Tcf transcription factors, and VDR. The epidermal phenotypes and Wnt/VDR transcriptional activity of wild-type and VDR–/– mice are compared in the presence or absence of *β*-catenin activation in 4OHT-treated K14-ΔN-*β*-catenin-ER transgenic mice. EF: ectopic hair follicle; BCC: basal cell carcinoma. TCF/Lef and VDR are shown bound to DNA in the presence of transcriptional corepressors (R) or *β*-catenin. The vitamin D ligand is indicated by an asterisk [40].

It is very important to consider the intersection between Wnt/*β*-catenin and other signaling pathways. There are two pathways interacting with Wnt/*β*-catenin signaling in the epidermis: Notch and vitamin D pathways. Notch1 plays an essential role in the development and homeostasis of postnatal hair follicles [36]. Wnt4 can be negatively regulated by p21 [37]; meanwhile, p21 is a direct transcriptional target of the Notch pathway. We suggest that the Notch pathway may affect the function of Wnt4 that is necessary for the development and homeostasis of postnatal hair follicles. The vitamin D receptor (VDR) is very important for hair maintenance, and the loss or mutation of VDR will result in hair loss in mice and humans [38]. For example, *β*-catenin can bind to and activate the vitamin D receptor (VDR) by cooperating with vitamin D response elements that significantly function in the postnatal maintenance of HFs [39, 40]. From the research of Watt and Collins, we can get a comprehensive view about interactions among *β*-catenin, TCF/Lef, and VDR. In wild-type epidermis, initiation of the growth phase in HF anagen is dependent on Wnt signaling activation. HFs are kept in the telogen phase in a mouse model with the functional loss of Wnt signaling. However, the cycling of hair growth was not maintained, and HFs were degenerated in the absence of VDR. A high level of Wnt signaling activation in the presence of endogenous vitamin D or a topically applied vitamin D analog not only was able to trigger anagen, but also induced ectopic formation of HFs. Wnt activation was unable to induce differentiation of ectopic follicles without the presence of VDR (Figure 2) [40].

In addition, one of the pathways that is upregulated by *β*-catenin activation in the epidermis is Sonic hedgehog (Shh) signaling [41]. Combined with the modest activation of *β*-catenin, pharmacological inhibition of Shh signaling can block* de novo* hair follicle formation. Conversely, a high level of *β*-catenin activation can make the inhibition of Shh signaling to improve morphogenesis [41]. The expression of Sox9 is dependent on Shh, and deletion of Sox9 in the epidermis can lead to hair loss and bulge forming failure [42], which results in the same outcome as the effects of blocking *β*-catenin signaling [43, 44]. Thus, we can reach the conclusion that *β*-catenin affects hair development via Shh, and Shh can function similarly to *β*-catenin. Therefore, we can target not only *β*-catenin but also Shh when we consider therapy for skin cancer.

## 4. Wnt/***β***-Catenin Signaling Pathway in Skin Cancer Stem Cells (CSCs)

Skin cancer is one of the most common forms of cancer with an especially high incidence in the white population [45]. There are many types of skin cancer, including squamous cell carcinoma, basal cell carcinoma, malignant melanoma, malignant lymphoma, idiopathic hemorrhagic sarcoma, fibrosarcoma, and sweat gland carcinoma.

Wnt/*β*-catenin signaling plays a critical role in skin CSCs. Recently, a population of CSCs was identified by Malanchi et al. These cells show similar molecular characteristics to normal stem cells in the bulge [46]. CD34+ bulge stem cells that reside in normal mouse skin account for nearly 2% of the keratinocytes. It is observed that there is nearly 10-fold increase of this type of CD34+ cell population in early skin tumors induced by the classical two-step chemical carcinogenesis protocol, DMBA and TPA, or by overexpression of mutant Ras (HRAS-R12T59). Furthermore, it has been identified that the ability of the CD34+ cells to induce secondary tumors is over 100-fold greater than that of unsorted cells. Therefore, it is clear that these CD34+ bulge stem cells indeed represent CSCs. In addition, the secondary tumors resemble the parental tumors and have maintained a small population of CD34+ CSCs. This discovery shows that there is a preferential location of *β*-catenin in the nucleus. Human squamous cell carcinomas also exhibit a preferential nuclear location of *β*-catenin. Remarkably, conditional ablation of *β*-catenin will result in complete regression of the tumors and terminal differentiation of the tissues through tamoxifen-induced mutagenesis in DMBA-TPA or Ras-induced tumors. Thus, Wnt/*β*-catenin signals are essential in normal skin to drive bulge stem cells toward the hair cell fate, whereas they play a critical role in the maintenance of skin CSCs in epidermal tumors [11].

## 5. Wnt/***β***-Catenin Signaling Pathway in Therapy of Skin Cancer

Because of drug resistance and the relapse and metastasis of cancer stem cells, skin cancer therapy is faced with a number of technical challenges and regulatory hurdles that must be overcome. In order to avoid all of these negative effects, it is very important to search for targeted and effective treatments for skin cancer. As described above, Wnt/*β*-catenin signaling contributes to the homeostasis of normal skin stem cells, and its malfunction results in production of skin cancer stem cells. Thus, we can prevent the generation of CSCs by regulating Wnt signaling specifically. Thereby, the therapy of skin cancer targeting Wnt/*β*-catenin signaling is an innovative strategy to cure skin cancer. Herein, several approaches will be introduced.

### 5.1. Inhibitors of Acyltransferase Porcupine

Porcupine (Porcn), a member of the membrane-bound O-acyltransferase (MBOAT) family, catalyzes the palmitoylation family of Wnt proteins [47]. Palmitoylation is a necessary process for the secretion and activity of Wnt. Compromised Porcn activity commonly results in developmental disorders including focal dermal hypoplasia (Goltz syndrome), whereas the hyperactivity of Porcn is associated with proliferation and metastasis of cancer cells. Constitutive activation of the Wnt signaling pathway is a feature of a number of cancers including malignant melanoma with aberrant nuclear accumulation and the subsequent upregulation of the *β*-catenin transcription response [48]. We can hypothesize that inhibition of Porcn could be the most effective strategy for broadly suppressing Wnt signaling. More importantly, Porcn inhibitors have proven to be remarkably nontoxic in rodents. Therefore, the inhibitors of Porcn hold great potential in the therapy of skin cancers with aberrant activation of Wnt signaling. The four IWPs (IWPs 1–4) (inhibitors of Wnt production), the first identified Porcn inhibitors, and LGK974, a novel Porcn inhibitor, have entered Phase I clinical trials.

### 5.2. Transcriptional Coactivator Antagonists

Several coactivators for Wnt/*β*-catenin transcription, such as CBP, p300, B-cell lymphoma 9 (BCL9), and pygopus, have been identified [49–51]. The lead compound ICG-001 that was selectively bound to CBP and prevented its interaction with *β*-catenin was identified using a cell-based reporter screen. While it is a small molecule secondary structure-template chemical library, ICG-001 does not interact with the homologous coactivator p300. Therefore, fundamental stem and progenitor cell switching points are regulated by the switch from *β*-catenin/CBP to *β*-catenin/p300 [52, 53]. Since CBP/*β*-catenin targets cellular proliferation and p300/*β*-catenin responds to cellular differentiation, the balance of CBP/*β*-catenin and p300/*β*-catenin may be very important for a cell to decide to either maintain its level of potency or go on to differentiate and lose a level of potency. Therefore, it may be an effective therapeutic strategy to find the inhibitors of CBP/*β*-catenin [54].

### 5.3. Nonsteroidal Anti-Inflammatory Drugs

NSAIDs, including aspirin, flurbiprofen, sulindac, and indomethacin, function by inhibiting the activity of cyclooxygenase (COX), which is a key enzyme in the arachidonic acid cascade. Recently, many experimental and epidemiological studies in humans have provided evidence that aspirin and other NSAIDs exhibit chemopreventive effects against multiple cancer types by inhibiting the Wnt/*β*-catenin signaling pathway. As for skin cancer, the Wnt/*β*-catenin pathway has been related to malignancy in nonmelanoma skin cancers (NMSCs) such as squamous cell carcinoma (SCC) and basal cell carcinoma (BCC) [55–57]. Previous evidence has demonstrated that NSAIDs have a chemopreventive effect against SCC and BCC [58]. The data suggested that long-term use (>5 years) of NSAIDs was related to a better protective effect of developing cutaneous melanoma [59]. Regular use of NSAIDs induces several serious side effects concerning the gastrointestinal, renal, and cardiovascular systems [60–62]. To overcome this defect, nitric-oxide-releasing NSAIDs (NO-NSAIDs), a new class of chemopreventive agents, were developed. They consist of a traditional NSAID attached to NO-releasing moiety through an aromatic spacer. Animal and human models have concluded that many NO-NSAIDs are safer for the gastrointestinal mucosa than the parent NSAID [63–66]. One example of this type of NO-NSAIDs is the newly synthesized flurbiprofen benzyl nitrate (FBN, NBS-242), which contains an aromatic spacer. Regarding the mechanism, FBN functions in skin cancer by inhibiting the growth of A-431 cells, affecting proliferation, and inducing apoptosis and targeting components of the Wnt pathway and inducing caspase-3. Except for this mechanism, several systematic reviews have suggested that NSAIDs function as a chemopreventive agent for patients that are predisposed to nonmelanoma skin cancer (NMSC) [45, 67].

### 5.4. Vitamin D

1,25-Dihydroxyvitamin D3 (1,25(OH)2D3), the active metabolite of vitamin D, suppresses the proliferation while promoting the differentiation of keratinocytes through the vitamin D receptor (VDR). However, *β*-catenin promotes proliferation and blocks epidermal differentiation although it stimulates hair follicle differentiation [68]. A recent study suggested that while 1,25(OH)2D3/VDR inhibits the actions of *β*-catenin to promote keratinocyte proliferation, 1,25(OH)2D3/VDR promotes the ability of *β*-catenin to stimulate hair follicle differentiation. The mechanism involved 4 aspects: 1,25(OH)2D3 and VDR suppress transcriptional activity of *β*-catenin/TCF, 1,25(OH)2D3 represses mRNA expression of the *β*-catenin target gene of Gli1 but induces the *β*-catenin target gene PADI1* in vitro*, VDR ablation reduces *β*-catenin target genes involved in hair differentiation* in vivo*, and 1,25(OH)2D3/VDR suppresses the transcriptional activity of *β*-catenin and represses the mRNA expression of Gli1 [69]. Recently it has also been identified that both vitamin A and vitamin D might induce Wnt/*β*-catenin inhibitory proteins; for instance, disabled-2 (Dab2) is induced by retinoic acids and Dickkopf-1 and Dickkopf-4 (Dkk-1 and Dkk-4) are induced by vitamin D [70, 71].

### 5.5. Natural Products

With the exception of chemical synthetic products, natural products, particularly traditional Chinese medicines (TCMs), are rich sources of pharmacologically active substances. Through the investigations, it has been discovered that stem cell signaling pathways could be targeted by the natural products [6]. Epidemiological evidence indicates that flavonoids that are rich in a plant-based diet are effective against cancer [72]. Fisetin (3,7,3′,4′-tetrahydroxyflavone) belongs to the flavonol subgroup of flavonoids and is found in many fruits and vegetables. Interestingly, the dietary flavonoid fisetin was observed to decrease cell viability of 451Lu melanoma cells by disruption of Wnt/*β*-catenin signaling. Fisetin-treated cells showed increased cytosolic levels of Axin and *β*-TrCP and decreased phosphorylation of glycogen synthase kinase 3*β* associated with decreased *β*-catenin stabilization. Fisetin-mediated interference with functional cooperation between *β*-catenin and T-cell factor- (TCF-) 2 resulted in the downregulation of positively regulated TCF targets, such as c-myc, Brn-2, and Mitf. Those data suggest that fisetin can be developed as an effective agent against melanoma because of its potential inhibitory effect on *β*-catenin/Mitf signaling [72].

### 5.6. Engineered Mouse Models (GEM)

Although mice rarely develop melanoma spontaneously, the genetically engineered mouse models of melanoma have been generated by different groups. Compared to xenograft hosts, mice used for GEM models have a fully functional immune system. Therefore, they serve as reliable and repeatable models to study the role of the immune system cells in melanoma biology and drug resistance [73, 74]. Dankort and colleagues developed a mouse melanoma model in which BRaf^V600E^ cooperates with Pten loss to induce metastatic melanoma [75]. This model provided a system to study features of melanoma metastasis and evaluate drugs for melanoma therapy, which could be used to prevent melanoma metastasis. The accumulating evidence shows that the Wnt/*β*-catenin signaling pathway is frequently upregulated in melanoma but its functional implication is unclear. Damsky and colleagues studied the functional role of *β*-catenin by modulating it in the BRaf^V600E^Pten−/− model. They showed that *β*-catenin is the mediator of melanoma metastasis to lymph nodes and lungs. In addition to its role in metastasis, *β*-catenin levels control cell differentiation and regulate both the MAPK and the PI3K/AKT signaling pathways [76]. This model is useful for drug screening of melanoma metastasis.

## 6. Conclusions

Stem cell research has a significant potential to enhance our understanding of cancer stem cells and thus revolutionize tumor therapy. Wnt/*β*-catenin signaling plays a critical role in both normal stem cells and CSCs. Over the past few years, remarkable progress has been achieved in research on Wnt/*β*-catenin signaling and its inhibitors. The pharmaceutical industry has placed high expectations on a number of clinical candidates that target Wnt signaling. There are many novel drugs and natural products targeting Wnt/*β*-catenin signaling pathway that have been developed.
